# Association between adipokines and thyroid carcinoma: a meta-analysis of case-control studies

**DOI:** 10.1186/s12885-020-07299-x

**Published:** 2020-08-20

**Authors:** Junyu Zhao, Jing Wen, Shengnan Wang, Jinming Yao, Lin Liao, Jianjun Dong

**Affiliations:** 1 Department of Endocrinology and Metabology, The First Affiliated Hospital of Shandong First Medical University & Shandong Provincial Qianfoshan Hospital, Ji-nan, 250014 China; 2 Department of Endocrinology and Metabology, Shandong Provincial Qianfoshan Hospital, Cheeloo College of Medicine, Shandong University, Ji-nan, 250014 China; 3 College of Traditional Chinese Medicine, Shandong University of Traditional Chinese Medicine, Ji-nan, 250000 China; 4 Department of Endocrinology and Metabology, Shandong First Medical University & Shandong Academy of Medical Sciences, Ji-nan, 250014 China; 5 Department of Endocrinology and Metabology, Qilu Hospital of Shandong University, Cheeloo College of Medicine, Shandong University, Ji-nan, 250012 China

**Keywords:** Thyroid carcinoma, Adipokines, TNF-α, IL-6, Leptin, Meta-analysis

## Abstract

**Background:**

The incidence of thyroid carcinoma is increasing all over the world. Some studies have suggested that the change of adipokines expression can induce thyroid carcinoma. However, other studies have come to the opposite conclusion. Therefore, we studied the relationship between adipokines and thyroid carcinoma.

**Methods:**

Databases—PubMed, Cochrane Library, SinoMed, CNKI, Wanfang, and clinical trial registries were searched. A meta-analysis was then performed through a fixed or random-effects model to calculate I values for heterogeneity analysis.

**Results:**

Twenty-nine articles were finally included for analysis. The level of serum tumor necrosis factor-alpha (TNF-α) [standardized mean difference (SMD) =1.31, 95% confidence interval (95% CI): 0.35 to 2.28, I^2^ = 98%, *P* = 0.008] and the ratio of TNF-α immunoreactivity in tissues [odds ratios (OR) =6.36, 95% CI: 1.92 to 21.05, I^2^ = 66%, *P* = 0.002] in thyroid carcinoma are significantly higher than those in control. The serum interleukin-6 (IL-6) in patients with thyroid carcinoma is higher than that in control (SMD = 1.04, 95% CI: 0.40 to 1.67, I^2^ = 96%, *P* = 0.001). There is no significant difference of the ratio of IL-6 immunoreactivity in tissues between carcinoma and control (OR = 1.23, 95% CI: 0.62 to 2.43, I^2^ = 86%, *P* = 0.55). The ratio of leptin immunoreactivity in tissues is significantly associated with the risk of thyroid carcinoma (OR = 12.21, 95% CI: 3.36 to 44.40, I^2^ = 85%, *P* < 0.00001). However, after analyzing the expression level of serum adiponectin in three studies, no significant difference is found between thyroid carcinoma and the control (*P* = 0.81).

**Conclusions:**

Adipokines (TNF-α, IL-6 and leptin) show a strong relationship between elevated concentrations (in serum and/or tissue) and thyroid carcinoma. However, the association between adiponectin and thyroid carcinoma needs further research.

## Background

Thyroid carcinoma is the most common endocrine malignancy but mostly has good prognosis. During the past decades, a rising incidence of thyroid carcinoma worldwide has aroused the widespread attention of researchers [1, 2]. Someone supposed that the growing use of diagnostic imaging and fine-needle aspiration biopsy may be the main reason [3]. But this may be only partial and can not totally explain the increased incidence of microcarcinoma. Changes in the incidence of a cancer are not only associated with increased detection and other unknown risk factors need further explore. Recently, some scientists found that the incidence of thyroid carcinoma has increased along with a marked rise in obesity rate, and accumulating evidence of an association between obesity and increased thyroid carcinoma risk has been proposed [4–6]. Various hypotheses have been supposed to interpret the relaitonship between obesity and thyroid carcinoma, including hyperinsulinemia, up-regulation of aromatase activity, chronic “low grade” inflammation, altered immune response, and DNA damage caused by oxidative stress [6]. Furthermore, recent data supporting the notion that a changed expression of adipokines caused by obesity can affect the cell proliferation and even induce a thyroid tumorigenesis [7–10]. Adipose tissue is a specialized connective tissue composed of fat cells which releases a number of biologically active molecules called adipokines (or adipocytokines), including leptin, adiponectin, resistin and many cytokines of the immune system, such as tumor necrosis factor-alpha (TNF-α), interleukin-6 (IL-6) and complement factor D (also known as adipsin). Adipokines refer to various enzymes, hormones, cytokines, growth factors, proteins and other biological active substances secreted by adipocytes, including adiponectin, leptin, resistin and interleukin. The concentration of adipokines, such as TNF-α, IL-6 and leptin, were significantly higher in obese subjects and the elevated levels was linked to obesity, and even positively correlated with body mass index [11–15]. It is reported that adipokines took part in the biological processes of insulin sensitivity, inflammation and proliferation [16, 17], which the proliferation have been recognized as an important factor leading to the tumorigenesis and development. At present, many kinds of adipokines have been reported to be associated with thyroid carcinoma. Rehem RA et al. [18] suggested that serum leptin levels were higher in well-deffierentiated thyroid carcinoma patients and a significant drop after surgery. Another envidence showed that adiponectin related with tumor size [19]. However, the opposite results were also found in other studies [20]. Some researches reported the expression of adipokines is lower in tumor tissue than normal control [21–23]. It is clearly that certain confounders, such as age, sex, ethnicity, and also heterogeneity in study size, methodology and original of sample, should be considered when trying to analyze the association between adipokines and thyroid carcinoma. These confunding factors above may be the cause of inconsistency results from different researches. Additionaly, the association between adipokines and thyroid carcinoma are still not well documented. Therfore, the aim of this meta-analysis was to investigate the association between adipokines and thyroid carcinoma, and propose that adipokine as a risk factor for thyroid carcinoma.

## Methods

### Searching progress

We conducted a search of all studies published until 27th July 2019, regarding the association between adipokine and thyroid carcinoma. Eligible case-control studies were found by searching the database of PubMed, Cochrane library, Sinomed, CNKI and Wanfang, and restricted to published results. Clinical trial register centers (http://www.clinicaltrials.gov) were also searched. The following search terms: (“Adipokine” or “Leptin” or “adiponectin” or “resistin” or “tumor necrosis factor-alpha” or “Interleukin-6” or “Complement factor D” or “Adipocytokines” or “tumor necrosis factor-α” or “TNF-α” or “IL-6” or “adipsin”) and (“thyroid cancer*” or “thyroid neoplasm*” or “thyroid tumor” or “thyroid carcinoma*” or “differentiated thyroid carcinoma” or “DTC” or “Papillary thyroid carcinoma” or “Thyroid carcinoma, papillary” or “PTC” or “Thyroid cancer, follicular” or “FTC” or “Thyroid Carcinoma, Anaplastic” or “ATC” or “Thyroid cancer, medullary” or “MTC”). Hand searching was used to identify appropriate studies including reference lists of eligible articles and related previous review articles. Eligible studies met the following criteria: (1) published in English or Chinese language; (2) study assessed the association between adipokine and thyroid carcinoma; (3) study designed as the case-control study; (4) study reported the expression of at least one adipokine either in blood or tissue. Studies were excluded if any of the followings were identified: (1) insufficient information concerning adipokine or thyroid carcinoma: outcome cannot directly extract or calculate OR and 95%CI, the type of study was not a case-control design, have not full-text; (2) animal trials.

### Study selection and data extraction

Two reviewers screened the studies and extracted data independently. Any disagreement was resolved by discussion or consensus with a third senior reviewer. Data included the following: first author, publication year, country; participant characteristics (i.e., mean age, sample size, sex ration, pathological type of thyroid carcinoma, source of controls); measured outcomes or the percentage of samples show immunoreactivity for adipokines antibody both in the case and control groups. The calculation method is shown below (take thyroid cancer for example): the number of samples obtained from thyroid carcinoma that show immunoreactivity for adipokines antibody divided by the total number of thyroid carcinoma samples).

### Statistical analysis

For meta-analysis, dichotomous outcomes were analyzed by using the odds ratios (OR) computed using the Mantel Haenszel method (fixed or random models). Continuous variables measured on the same scale, expressed as a mean value and standard deviation, were analyzed by using weighted mean differences (WMD). Otherwise, standardized mean difference (SMD) were used for different scale. All results were reported with 95% confidence interval (95% CI). I^2^ was used to assess heterogeneity between studies, and I^2^ values of 0, 25, 50 and 75% representing no, low, moderate and high heterogeneity, respectively. Visual inspection of the funnel plot was done to assess publication bias. The analyses were performed by Review Manager 5.3 (Cochrane Collaboration, United Kingdom, http://www.cochrane.org).

### Quality assessment and risk of bias

The methodological quality of case-control study was assessed by the Newcastle-Ottawa Scale (NOS) (Supplement Table 1), which consists of the three parameters (eight questions with nine possible scores): Selection, Exposure and Comparability. A study can be awarded a maximum of one score for each numbered item within the Selection an Exposure categories. A maximum of two scores can be got for Comparability. A higher score means better quality in methodology and five or more scores were considered to be of high quality. Disagreements were resolved by reevaluating and discussing between two reviewers.

## Results

### Search results and characteristics of included studies

**Fig. 1:**
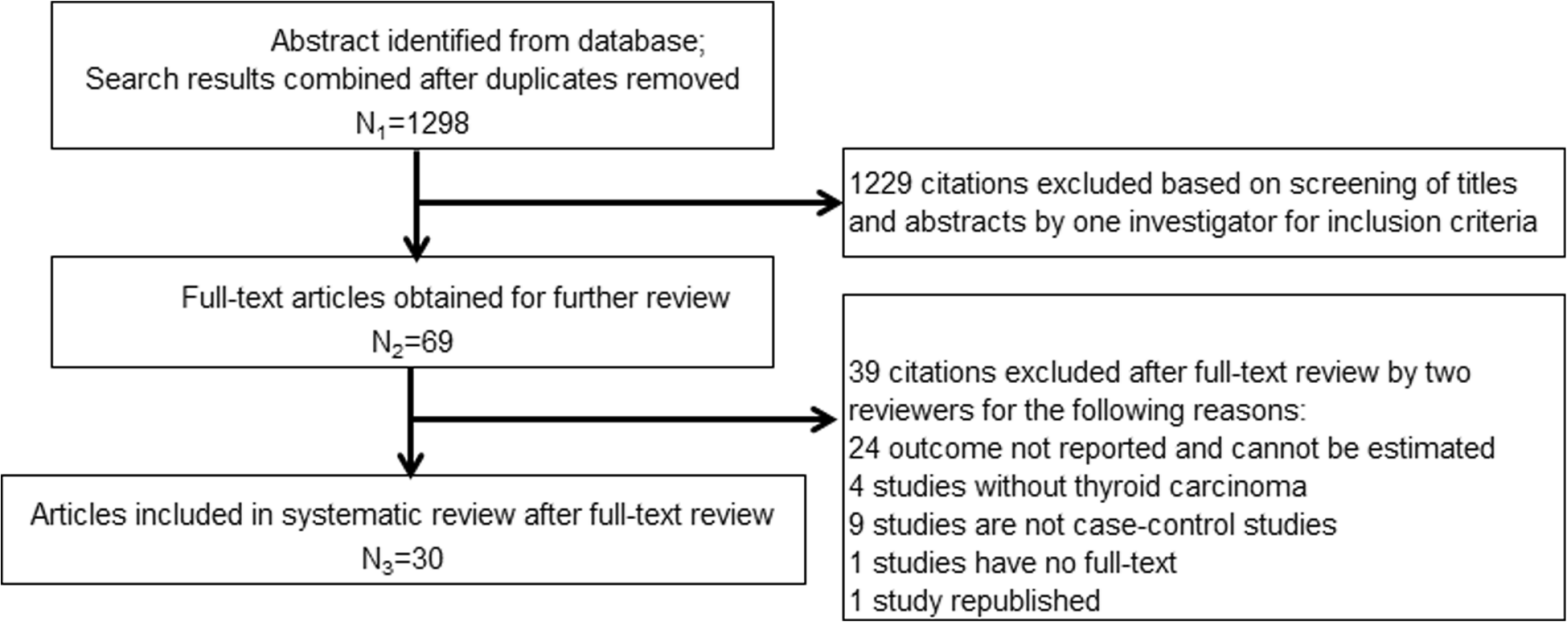
Flow chart of the systematic search process

1298 articles, regarding the association between adipokine and thyroid carcinoma, were searched in the related database and clinical trial websites. After screening the title and abstracts, 69 articles were selected for full-text review. Finally, 30 studies were eligible in this meta-analysis. Searching progress, included and excluded details are all shown in Fig. 1. Eighteen of these 30 studies are published in Chinese [21, 22, 24–39] and the rest are published in English [40–49]. Nineteen studies were conducted in China, two in India and two in Turkey. Brazil, Greece, Iran, Italy, Denmark and Serbia each had one study. Totally, there are 2174 patients with thyroid carcinoma in the case group and 1807 controls including healthy subjects, patients with benign thyroid diseases or normal thyroid tissue near carcinoma were included in the control group. The sample size ranges from 10 to 236 in the case group while 13 to 131 in the control group. All the thyroid carcinoma patients were confirmed by pathologically. Among these 30 studies, fourteen studies reported papillary thyroid carcinoma (PTC), eight studies reported differentiated thyroid carcinoma (DTC), three studies reported different pathological types in one paper, one study reported medullary thyroid carcinoma (MTC), and the rest four studies did not show the pathological details. The detailed characteristics of included studies are summarized in Table 1.

**Table 1:** Characteristic of 30 included studies

First author, Year	Country	Pathological type of thyroid cancer	Source of controls	Number of participants, n		Mean age, year		Female (%)		Outcome index
				cases	control	cases	control	cases	control	
L. Kayser, 1996 [33]	Denmark	PTC and FTC	multinodular goiters, adenomas, Hashimoto’s thyroiditis, hyperplastic glands	10	29	Unknown		Unknown		TNF-α (+) %--tissue
Cao Guangyao, 1998 [24]	China	Unknown	thyroid adenoma and nodular goiter	44	27	Unknown		Unknown		TNF-α (+) %--tissue
M.Trovato, 2003 [23]	Italy	DTC and undifferentiated carcinoma	normal thyroid tissues and benign nodules	28	46	Unknown		Unknown		IL-6 (+) %--tissue
Zhao Jianqiang, 2007 [25]	China	PTC, FTC, ATC and MTC	thyroid adenoma and normal health	236	131	Unknown		Unknown		IL-6、TNF-α--blood
Melih Akinci, 2009 [41]	Turkey	PTC	healthy volunteers	43	30	42.8 ± 13.2	54.6 ± 8.9	100%	100%	leptin--blood
Wang Jingxia, 2009 [26]	China	PTC and FTC	normal thyroid tissues	62	18	Unknown		87.10%	Unknown	TNF-α (+) %--tissue
Zhuang Xiaoming, 2010 [27]	China	PTC, FTC and MTC	thyroid adenoma and normal health	38	100	46	46/48	73.70%	Unknown	IL-6、TNF-α--blood
Yu Xiao, 2011 [28]	China	PTC	thyroid adenoma and normal thyroid tissue near carcinoma	58	26	Unknown		Unknown		leptin(+)%--tissue
Hou Sen, 2013 [29]	China	PTC	thyroid adenoma	76	16	Unknown		73.70%	Unknown	leptin(+)%--tissue
Snezana Zivancevic-Simonovic, 2014 [42]	Serbia	WDTC	healthy subjects	13	13	51.23 ± 14.9	45.75 ± 12.89	84.60%	84.60%	TNF-α--blood
Xu Xiaocheng, 2014 [30]	China	thyroid carcinoma	thyroid adenoma	44	36	54.3 ± 18.6	58.4 ± 17.4	36.40%	55.60%	IL-6--blood
Xeni Provatopoulou, 2014 [43]	Greece	PTC	benign thyroid *d*isease and healthy controls	20	38 + 50	49.2 ± 13.7	48.9 ± 14.5 / 49.5 ± 13.2	80%	81.6% / 86.0%	IL-6--blood
Sun Qinnuan, 2014 [31]	China	PTC	normal thyroid tissue near carcinoma and healthy controls	74	74 + 26	40.3 ± 3.6	40.3 ± 3.6 / 37.9 ± 2.4	60.81%	60.81% / 53.85%	TNF-α--blood and tissue
Zhang Zijie, 2014 [32]	China	PTC	thyroid adenoma	60	20	Unknown		73.33%	Unknown	leptin(+)%--tissue
Zhong Xiuxiu, 2014 [33]	China	PTC	thyroid adenoma	78	12	Unknown		Unknown		adiponectin(+)% --tissue
Zhang Bo, 2014 [34]	China	DTC	normal thyroid tissue near carcinoma	167	40	Unknown		82.63%	Unknown	adiponectin--tissue
Hu Jinhua, 2015 [35]	China	DTC	thyroid adenoma and healthy controls	64	42 + 40	49.8 ± 9.1	36.8 ± 11.3 / 45.3 ± 8.1	75%	69.04% / 70%	IL-6、 TNF-α--blood
Snezana Zivancevic-Simonovic, 2015 [44]	Serbia	PTC	control subjects	16	24	Unknown		Unknown		IL-6--blood
Yan-Lan Fan, 2015 [45]	China	thyroid carcinoma	nodular goitre, Hashimoto’s thyroiditis, follicular adenoma and adjacent non-neoplastic thyroid tissue samples	173	162	Unknown		Unknown		leptin(+)%--tissue
Wang Xinzheng, 2015 [36]	China	thyroid carcinoma	benign thyroid disease and normal thyroid tissue near benign thyroid disease	40	40 + 40	72.35 ± 7.44	72.83 ± 7.73	40%	35% / 35%	TNF-α--tissue
Song Runbo, 2015 [37]	China	PTC	thyroid adenoma	60	60	40.5 ± 8.4	46.7 ± 5.6	60%	53.33%	TNF-α (+) %--tissue
Kemal Beksac, 2016 [46]	Turkey	PTC	healthy volunteers	31	39	44	41	100%	100%	IL-6--blood
Toral P. Kobawala, 2016–1 [47]	India	PTC	benign thyroid diseases and healthy individuals	83	67 + 67	Unknown		67.47%	Unknown	TNF-α--blood
Toral P. Kobawala, 2016–2 [48]	India	PTC	benign thyroid diseases and healthy individuals	84	67 + 67	Unknown		67.47%	Unknown	IL-6--blood
Raziyeh Abooshahab, 2016 [20]	Iran	MTC	healthy subjects	45	45	29.46 ± 13.97	27.53 ± 13.66	53.33%	46.67%	leptin、 adiponectin--blood
Zhang Bo, 2016 [38]	China	DTC	normal thyroid tissue near carcinoma	167	40	Unknown		82.63%	Unknown	leptin--tissue
Zhou Xiaodong, 2016 [39]	China	DTC	healthy subjects	50	50	43.82 ± 12.58	42.96 ± 13.29	56%	52%	IL-6、TNF-α--blood
Ma Xiaokai, 2016 [22]	China	PTC	thyroid adenoma	60	45	Unknown		58.33%	Unknown	leptin(+)%--tissue
Mariana Bonjiorno Martins, 2017 [49]	Brazil	DTC	benign thyroid nodules and healthy controls	200	60 + 100	40.73 ± 13.88	47.95 ± 14.17 / 40.35 ± 13.34	86.50%	91.67% / 82%	IL-6--blood
Sun Zhenhua, 2017 [21]	China	PTC	nodular goiter	50	20	41.2	43.1	64%	70%	IL-6 (+) %--tissue

*TNF-α* tumor necrosis factor-a, *DTC* differentiated thyroid carcinoma, *IL-6* interleukin-6, *PTC* papillary thyroid carcinoma, *FTC* follicular thyroid carcinoma, *ATC* anaplastic thyroid carcinoma, *MTC* medullary thyroid carcinoma, *WDTC* well-differentiated thyroid carcinoma, *FNAC* fine needle aspiration cytology

### Quality of included studies

The quality assessment of these 30 studies is assessed by the NOS and the result is shown in Supplemental Table 2. Five or more scores are determined as high quality. Two studies conducted by Cao G et al. in 1998 [24] and L. Kayser et al. in 1996 [40] only get two scores showing a poor quality in methodology. The rest 28 studies are assessed as high quality.

### TNF-α and thyroid carcinoma

**Fig. 2:**
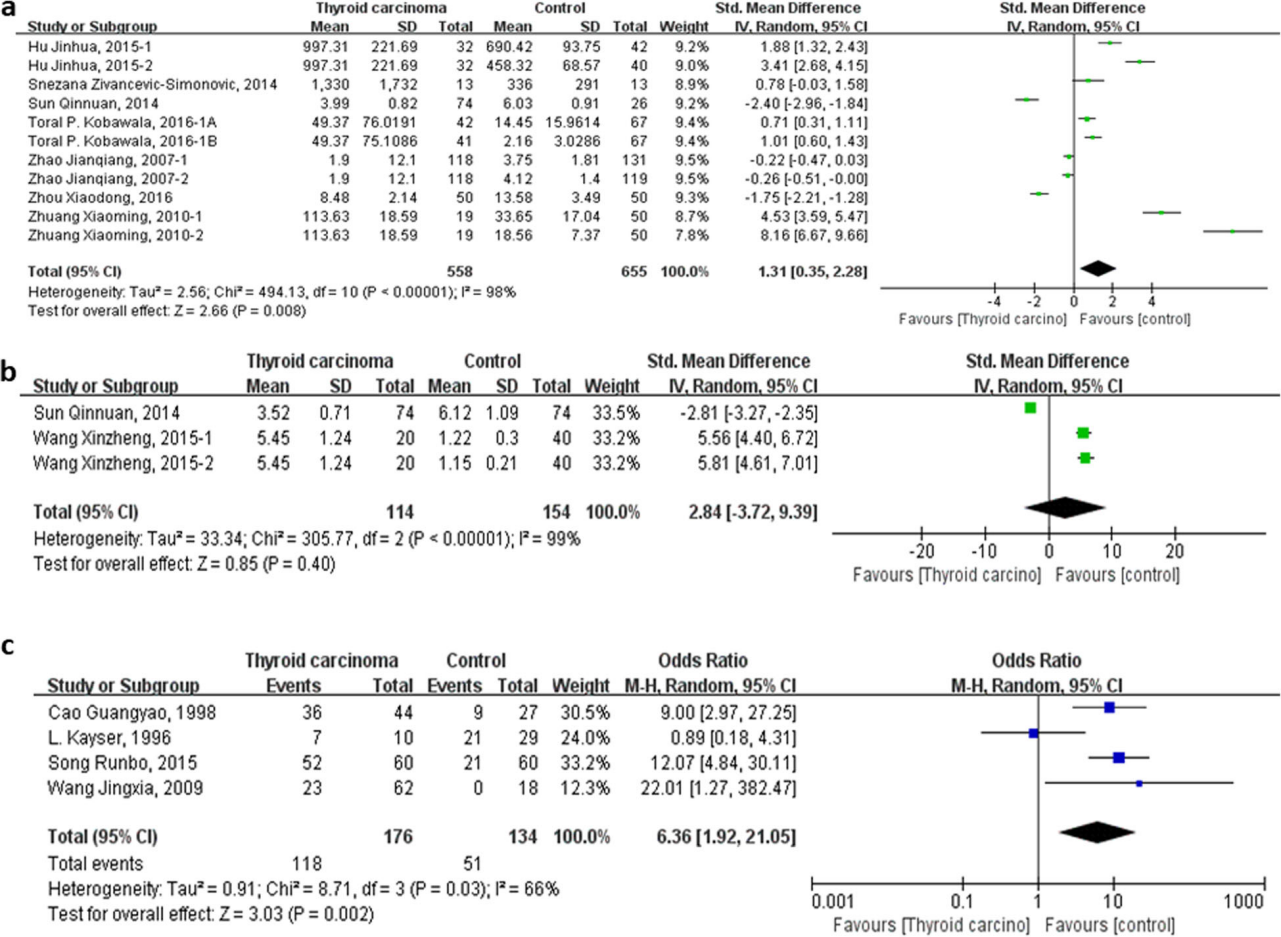
Forest plot of the TNF-α level and the ratio of TNF-α immunoreactivity in tissues in patients with thyroid carcinoma. a Level of serum TNF-α. b Expression of TNF-α in tissue. c Ratio of TNF-α immunoreactivity in tissue

Twelve studies reported the expression of TNF-α both in patients with thyroid carcinoma and control subjects [24–27, 31, 35–37, 39, 40, 42, 47]. Among these, seven studies [25, 27, 31, 35, 39, 41, 46] had tested the level of serum TNF-α, two studies [31, 36] had tested the expression of TNF-α in tissues, and the ratio of TNF-α immunoreactivity was tested in four studies [24, 26, 37, 40]. Firstly, fixed-effect model is used to merge the SMD values of serum TNF-α level, however, a large heterogeneity is found by the heterogeneity analysis (heterogeneity test, Chi^2^ = 494.13, *P* < 0.00001, I^2^ = 98%) and it may be due to the different units, different testing methods in different researches, or other unknown factors. Then, random-effect model to merge the SMD is used and pooled effect size in favor of control group is 1.31 (95% CI 0.35 to 2.28, *P* = 0.008) (Fig. 2a). SMD values of the expression of TNF-α in tissues is merged by fixed-effected model and the heterogeneity analysis show a considerable heterogeneity (heterogeneity test, Chi^2^ = 305.77, *P* < 0.00001, I^2^ = 99%). The different units and limited numbers of research may be the original of heterogeneity. So, the pooled SMD with random-effect model of the expression of TNF-α in tissues is 2.84 (95% CI − 3.72 to 9.39, *P* < 0.00001) (Fig. 2b). The pooled OR with fixed-effect model of the ratio of TNF-α immunoreactivity in thyroid carcinoma tissues is 7.67 (95% CI 4.11 to 14.31, *P* < 0.00001). However, a significant heterogeneity is detected (heterogeneity test, Chi^2^ = 8.71, *P* = 0.03, I^2^ = 66%). The article published by L. Kayser in 1996 with a poor quality in methodology may attribute to this high heterogeneity. Then, random-effect model of pooled OR is used and pooled effect size in favor of control group is 6.36 (95% CI 1.92 to 21.05, *P* = 0.002) (Fig. 2c). In conclusion, level of serum TNF-α and the ratio of TNF-α immunoreactivity in tissues of thyroid carcinoma patients are significantly higher than control subjects which are without thyroid carcinoma.

### IL-6 and thyroid carcinoma

**Fig. 3:**
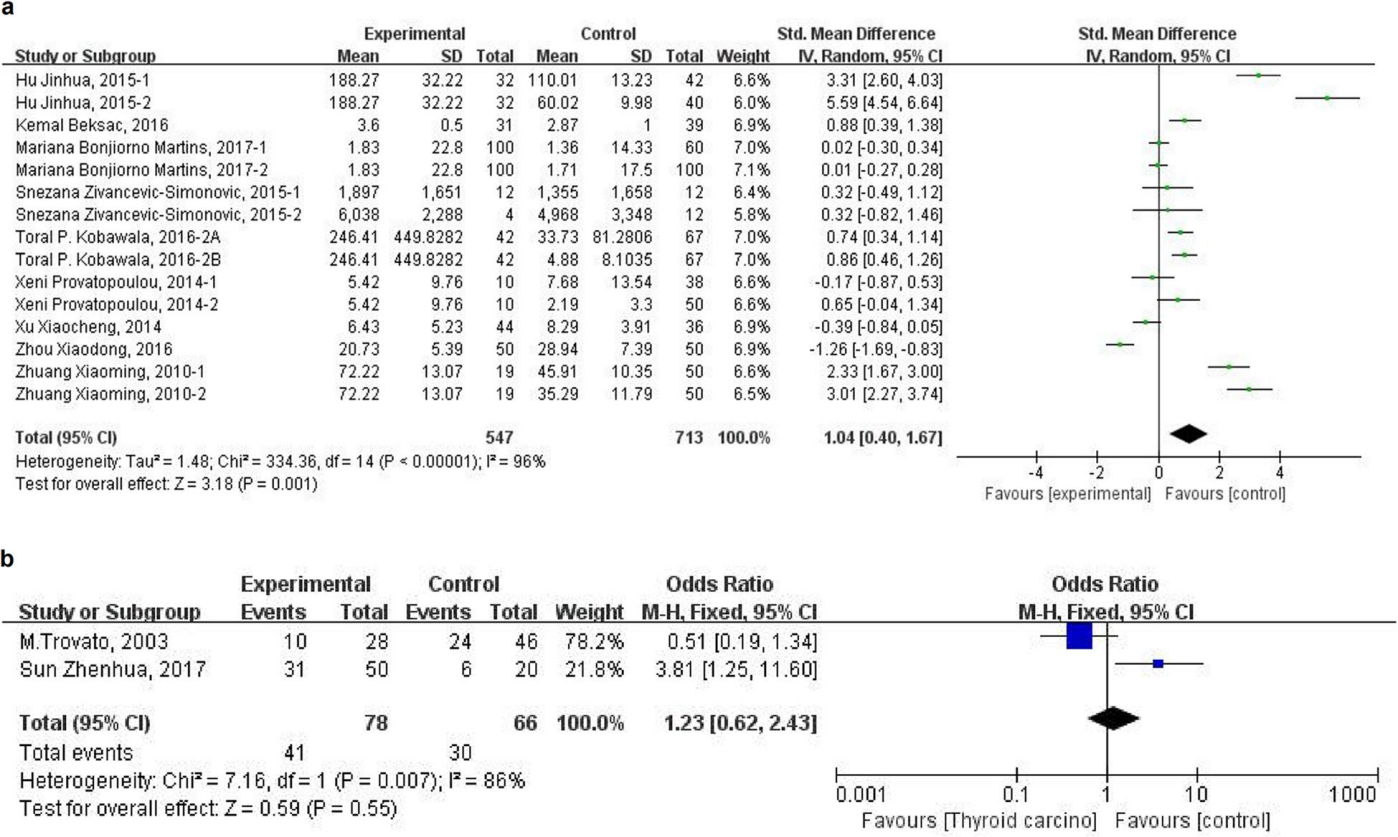
Forest plot of the IL-6 level and ratio of IL-6 immunoreactivity in tissue in patients with thyroid carcinoma. **a** Level of serum IL-6. **b** Ratio of IL-6 immunoreactivity in tissue

Among the 30 included studies, 9 reported the level of serum IL-6 in patients with thyroid carcinoma and control subjects [27, 30, 35, 39, 43, 44, 46–49]. Due to the large heterogeneity of the merged SMD values of serum IL-6 level by the heterogeneity analysis (heterogeneity test, Chi^2^ = 334.36, *P* < 0.00001, I^2^ = 96%), random-effect model was used to pooled the SMD values, and the pooled effect size in favor of control subjects is 1.04 (95% CI 0.40 to 1.67, *P* = 0.001) (Fig. 3a), which means that patients with thyroid carcinoma have a significantly higher level of serum IL-6 than control subjects. Two studies reported the ratio of IL-6 immunoreactivity both in thyroid carcinoma tissue and non-carcinoma tissue [21, 23]. The pooled OR of the limited two studies do not show an increased ratio of IL-6 immunoreactivity in thyroid carcinoma tissues (OR = 1.23 (95% CI 0.62 to 2.43, *P* = 0.55)) and a large heterogeneity always exists (heterogeneity test, Chi^2^ = 7.16, *P* = 0.007, I^2^ = 86%) (Fig. 3b). Thus, the level of serum IL-6 is higher in patients with thyroid carcinoma. However, it needs more clinical data to verify the relationship between the expression of IL-6 and thyroid carcinoma tissue.

### Leptin and thyroid carcinoma

**Fig. 4:**
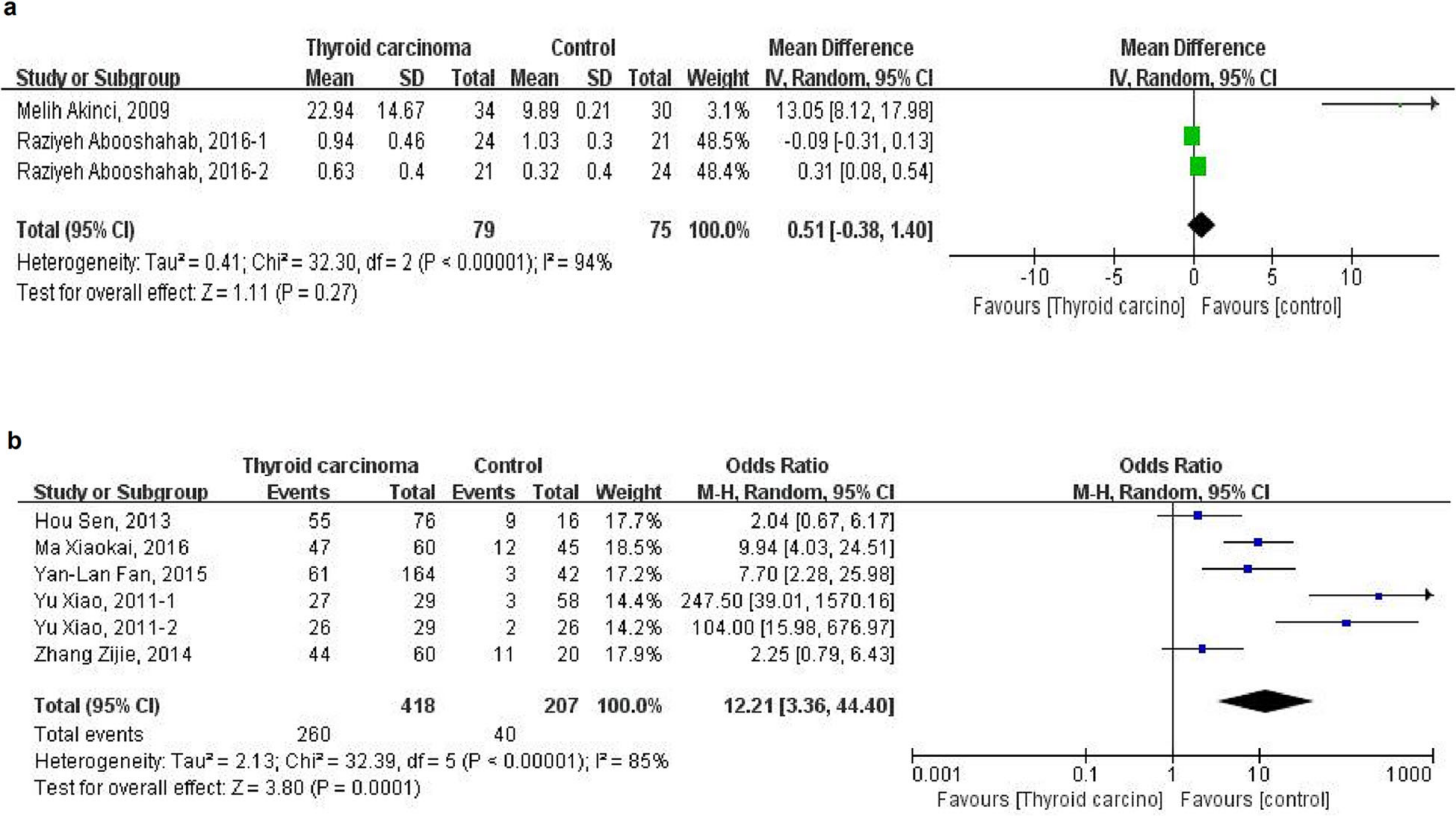
Forest plot of the leptin level and ratio of leptin immunoreactivity in tissuein patients with thyroid carcinoma. **a** Level of serum leptin. **b** Ratio of leptin immunoreactivity in tissue

Two studies reported the level of serum leptin [20, 40] and another five studies reported the ratio of leptin immunoreactivity in tissues [22, 28, 29, 32, 45]. Because of the considerable heterogeneity of the pooled WMD of serum leptin level (heterogeneity test, Chi^2^ = 32.30, *P* < 0.00001, I^2^ = 94%) and pooled OR of the ratio of leptin immunoreactivity in tissues (heterogeneity test, Chi^2^ = 32.39, *P* < 0.00001, I^2^ = 85%) by the heterogeneity analysis with fixed-effect model, random-effect model is further used to merge the values and analysis. However, there is no association of higher level of serum leptin with risk of thyroid carcinoma (WMD = 0.51, 95%CI (− 0.38 to 1.40)) (Fig. 4a). Moreover, the pooled OR of the ratio of leptin immunoreactivity in tissues from five studies is 12.21 (95%CI 3.36 to 44.40) (Fig. 4b), which means a high ratio of leptin immunoreactivity in tissue is significantly related to thyroid carcinoma.

### Adiponectin and thyroid carcinoma

Three studies reported the expression of adiponectin in thyroid carcinoma, including serum and tissue [20, 33, 34], and the result is summarized in Table 2. It could be found that the level of serum adiponectin is not statically different comparing thyroid carcinoma patients with control subjects (*P* = 0.81). Interestingly, it was found that the expression of adiponectin in thyroid carcinoma tissue is significantly lower than control tissue, while the opposite result is found when comparing the ratio of adiponectin immunoreactivity. However, there was only one study for each result and this may be the reason why the two results are diametrically opposed. Thus, it needs more clinical studies to confirm in the future.

**Table 2:** Summary of adiponectin expression in thyroid carcinoma

	Effect size	95%CI	P	I^2^
serum adiponectin [20]	WMD = 0.01	−0.05, 0.07	0.81	0%
ratio of adiponectin immunoreactivity [33]	OR = 6.00	1.39, 25.86	0.02	Not applicable
adiponectin in tissue [34]	WMD = -4.35	−4.64, −4.05	< 0.00001	99%

*95% CI* 95% confidence interval, *WMD* weighted mean differences, *OR* odds ratios

### Publication bias

**Fig. 5:**
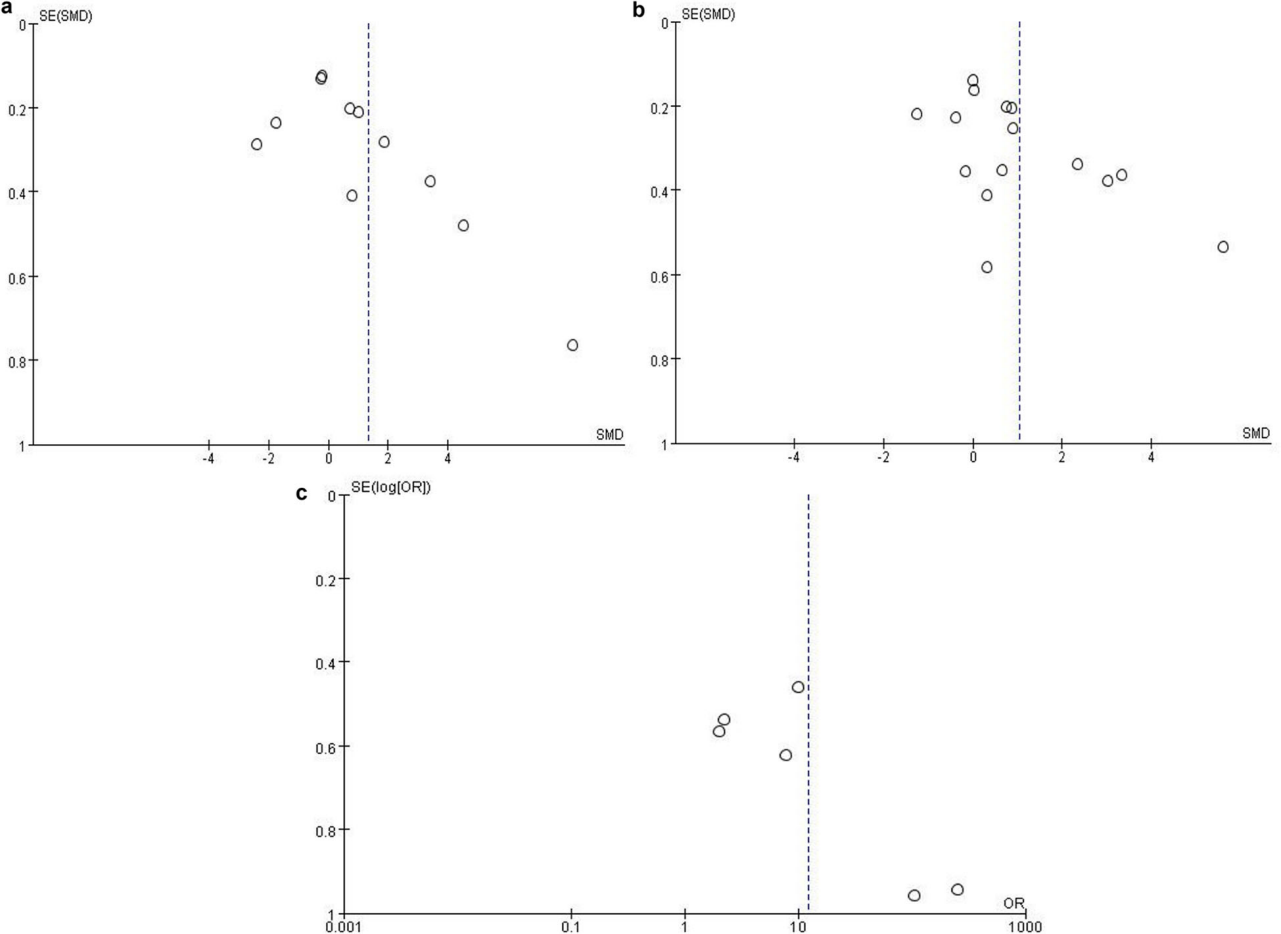
Funnel plots of **a** TNF-α, **b** IL-6 and **c** leptin revealed no significant publication bias. *SE (SMD)* standard error of standardized mean difference

The funnel plot was applied for assessing publication bias of studies included in the three results, including TNF-α (Fig. 5a), IL-6 (Fig. 5b) and leptin (Fig. 5c). In Fig. 5a and Fig. 5b**,** almost all studies lies inside the 95%CIs, with an even distribution around the vertical, indicating no evident publication bias was obtained through the visual distribution of funnel plot. However, a potential publication bias was found in Fig. 5c when comparing the ratio of leptin immunoreactivity in tissues, and that might influence the result of this meta-analysis.

## Discussion

Currently, obesity affects one third of population among US adults [50], and China has become a big country of obesity with the incidence ranking first worldwide in the year of 2014 [51]. Nowadays, increasing clinical and experimental studies and documented the closely relationship between malignancies (including colon, esophagus, kidney, liver, breast, endometrium, pancreas and prostate as well as non-Hodgkin’s lymphoma and multiple myeloma) and obesity/overweight, which affect its occurrence, development and prognosis [52–54]. Because of the increasing incidence of thyroid carcinoma during the past decades, lots of scientists focus on studying the risk factors of thyroid carcinoma. It was found that the incidence of thyroid carcinoma has increased along with a marked rising rate of obesity [4–6]. Furthermore, obesity is an independent risk factor for thyroid carcinoma [55]. Increased insulin resistance, elevated serum cholesterol level and upregulated COX2 expression may be the target of the correlation between obesity and thyroid carcinoma [56]. It is reported that people with higher body mass index have a higher concentration of adipokines [12–16]. Adipokines take part in the following pathological and physiological processes, such as, insulin sensitivity, inflammation and proliferation [17, 57], and these are important in the process of tumorigenesis and developing. So adipokines may be one of the targets linking obesity with thyroid cancer. The meta-analysis was based on previous published studies. In previous studies, the analysis of adiponectin and thyroid cancer mostly focused on TNF-, IL-6, Leptin and Adiponectin. While few studies focused on other molecules (including IL-1 and IL-8) and we failed to combine statistics. Therefore, in this meta-analysis, only TNF-, IL-6, Leptin and Adiponectin, which are the most published adiponectin, were analyzed.

TNF-α, produced by adipose tissue and inflammatory cells, can lead to inflammatory response, necrocytosis, and assist other cytokines to kill tumor cells, and improve the anti-tumor ability. Meanwhile, TNF-α plays an important role in the process of inflammation, insulin resistance, diabetes and obesity. A moderate amount of TNF-α has a protective effect, while an excessive amount will cause damage, which may lead to a resistant of tumor cells to TNF-associated apoptosis-induced ligands when the microenvironment of apoptosis is maladjusted. TNF-α has the ability to promote the production of granulocyte-colony stimulating factor by thyroid fibroblasts [58], which may play an important role in thyroid cancer. Moreover, TNF-α can stimulate the vasoactive mediators such as interleukin and prostaglandin [59], and these mediators can promote the proliferation of tumor cells and significantly reduce the immune function. TNF-α can also induce an increased expression of vascular endothelial growth factor (VEGF) [60], the later of that can promote the proliferation of tumor cells and provide conditions for tumors metastasis.

In conclusion, surprisingly, the results of clinical studies provide evidence for basic research. Simonovic SZ et al. [42] evaluated cytokine profiles (determined in supernatants obtained from whole blood cultures) in 13 patients with DTC before and 7 days after radioactive iodine (131-I) therapy and 13 control subjects, and found that the expression of TNF-α in DTC patients is higher than control subjects, and it showed a decreased level after 131-I therapy than those before therapy. However, no statistical difference found for the limited sample size. Another study conducted by Kobawala TP et al. [47], with more patients (67 patients with benign thyroid disease, 83 PTC patients and 67 healthy individuals), determined the circulating levels of TNF-α, and it was found that the serum level of TNF-α was significantly higher in PTC patients than benign thyroid disease patients, and the later was also significantly higher than healthy individuals. Furthermore, serum TNF-α was reported to be a significant prognosticator for overall survival in PTC patients. It is a pity thatopposite result was reported in a case-control study that included 475 DTC cases and 1016 matched cancer-free cohort participants, which found that TNF-a was not associated with thyroid risk in either gender [61].

Based on current evidence, our meta-analysis suggests that TNF-α exhibit a strong association with thyroid carcinoma. It may because that elevated TNF-α may involved in the tumorigenesis and development of thyroid cancer. Another possible reason is that the TNF-α decreased with tumor cells less resulted the activation of the immune system by thyroid carcinomaTherefore, more clincal studies and basic reseaches should be conducted in the future.

IL-6, a multifunctional cytokine, plays important roles in different types of cells including tumor cells. It is reported that elevated serum IL-6 level is closely related to the tumorigenesis and development of a variety of tumors [62]. A strong positive association between the serum IL-6 and the progression and poor prognosis of tumors in patients with several types of tumor was already found [63–65]. Serum IL-6 level in thyroid cancer has been evaluated in numerous studies including in vivo and in vitro studies. Provatopoulou X et al. [43] found that serum IL-6 were significantly higher in malignant and benign thyroid diseases compared to healthy controls. However, other studies show a different result that no significance different of IL-6 was found between thyroid cancer and non-thyroid cancer [16, 23, 43, 44, 49]. A limited sample size, different inclusion criteria, different population characteristics, or different pathological type of thyroid cancer may explain such a difference. For in vitro research, IL-6 was also found to be expressed in thyroid cancer cell lines and a potential role of IL-6 in PTC was confirmed indirectly [66].

The underlying mechanism may be the followings below. Tumor cells including esophageal cancer, lung cancer, colorectal cancer and melanoma were found have the function of autocrine IL-6, which can affect the growth and proliferation of tumor cells and participate in the tumor growth and metastasis by acting on the membrane receptors [67]. Also, IL-6R was found associated with the characterization of thyroid nodules’ malignancy and tumor aggressiveness [49]. In addition, Iliopoulos D et al. [68] found that Src (non-somatic tyrosine kinase family oncogene) can induce the normal epithelial cell transformation by activating NF-κB, and this transformation contributes to tumorigenesis. IL-6 is considered as an important regulatory factor in this process. Another possibility is that the activation of the immune system of patients with thyroid cancer leads to an increase in adikopines level.

In general, the data above support that IL-6 is important for thyroid cancer, but the detail mechanism remain to be further study.

Leptin, a circulating hormone secreted by adipocytes, exerts its biological effect by combing with its receptor, which is mainly presented in the hypothalamus. Meanwhile, gene of leptin receptor is also expressed in many other tissues, such as lung, liver and kidney. It is reported that obesity and overweight can lead to a high level of serum leptin, which may because that obesity always accompanies with insulin resistance and hyperinsulinemia, and insulin further enhance the expression of leptin. Moreover, leptin acts as a growth factor in a variety of human cells, including both normal cells and tumor cells, which regulates the process of differentiation, proliferation and apoptosis thus stimulate the tumorigenesis and development of tumors through mediating JAK/STAT3 pathway, RhoA/LIMK1/Cofilin pathway, and MAPK/ERK pathway, [69]. Kim WG et al. [70] evaluated the effect of diet-induced obesity on thyroid carcinogenesis in a mouse model that spontaneously develops thyroid cancer (Thrb (PV/PV) Pten (+/−) mice) and found that obesity increases the frequency of anaplasia of thyroid cancer and exacerbates thyroid cancer progression that were mediated by increased activation of the JAK2 signaling transducer and activator of STAT3 signaling pathway and induction of STAT3 target gene expression. Leptin is always reported a high expression on solid tumors [71], and it is confirmed that serum leptin level is significantly increased in thyroid cancer (mainly PTC), while other studies showed a same results in cancer tissues [11, 15, 21, 41, 45]. Yu Xiao et al. [21] conducted a clinical study comparing the level of serum leptin in 58 PTC patients (including 29 patients with lymph node metastasis) and 26 thyroid adenoma patients in Dalian, China, and found that patients with lymph node metastasis have a higher level of leptin than those without lymph node metastasis. Leptin can induce the expression of vascular endothelial growth factor and promote neovascularization in tumor tissue [72]. In addition, it can also inhibit the apoptosis through Bcl-2 dependent mechanism. Meanwhile, leptin receptor exists in all thyroid cancer cells. It is overexpressed in PTC and is involved in tumor invasion and lymph node metastasis [73, 74]. Thus, leptin may be involved in the tumorigenesis and metastasis of thyroid cancer through a complex pathway and a monitoring may have some significance. Due to the absence of direct evidence, elevated leptin levels can also be caused by thyroid carcinoma. The cause and effect relationship between leptin and thyroid carcinoma are unclear now and need further studies.

Compared to lean women, overweight/obese women had lower serum adiponectin levels and this difference has statistical significance [75]. In addition, adiponectin is negatively associated with a variety of benign and malignant tumors, especially those associated with obesity and insulin resistance, such as leukemia [76], renal carcinoma [77], gastric carcinoma [78] and colon cancer [79]. Moreover,, the association of adiponectin with potential tumor-limiting functions has been widely proposed [80].

Otvos L Jr. et al. [81] tried in vitro experiments and proved that adiponectin can inhibit the metastasis of cancer cells. Mitsiades N et al. [82] measured circulating adiponectin levels in ptaients with PTC and found that it is independently and inversely associated with the risk of thyroid cancer. As the receptor that binds to adiponectin for biological effects, adiponectin receptor had been reported closely correlated with the development of PTC. Adiponectin receptor-1 and 2 are higher expression in PTC tissues than that in the surrounding normal tissues and this is thought to be associated with a better prognosis [83].

However, other studies have shown different results [13, 27] and more studies should be done furtherly to support the anti-tumor effect of adiponectin, and the positive correlation between the increased level of adiponectin in circulating blood and the prognosis of thyroid neoplasms and provide new ideas for the prevention and treatment of thyroid neoplasms.

From the above, a strong relationship between elevated concentrations of adipokines (in serum and/or tissue) and thyroid cancer can be concluded. And this may explain why increased incidence of obesity and thyroid cancer are consistent. Thus, targeted drugs for adipokine may be useful for the treatment of thyroid cancer in the future.

However, some limitations in our meta-analysis should be taken into account. First, some data were not normally distributed and were reported in the form of median and quartile, and therefore these data were calculated by formulas. Second, due to the insufficient database access, six articles are not available in full, and therefore could not be included in this meta-analysis. Third, all the included studies were cross-sectional case-control study and the dynamic changes of these adipokines in preoperative and postoperative were not provided. The last but not the least, most of the included studies (18 of these 30 studies) are published in Chinese, thus a considerable but may inevitable bias can result of this meta-analysis. All these limitations above should be improved in the future study, thus a strong conclusion could be get.

## Conclusions

In summary, our meta-analysis suggests that adipokines, including TNF-α, IL-6 and leptin are associated with thyroid carcinoma. Nevertheless, it is not conclusive for adiponectin due to the limited number of the clinical studies. Therefore, larger sample sizes of different ethnic population are required to confirm and update our findings.

## Supplementary information

**Additional file 1 Supplemental Table 1** Newcastle-Ottawa Quality Assessment Scale—Case-control Studies.

**Additional file 2 Supplemental Table 2** Quality assessment according to the Newcastle-Ottawa Scale.

## Data Availability

The datasets used and/or analyzed during the current study are available from the corresponding author on reasonable request.

## Abbreviations

TNF-α: Tumor necrosis factor-alpha
IL-6: Interleukin-6
OR: Odds ratios
WMD: Weighted mean differences
SMD: Standardized mean difference
95% CI: 95% confidence interval
NOS: Newcastle-Ottawa Scale
DTC: Differentiated thyroid carcinoma
PTC: Papillary thyroid carcinoma
FTC: Follicular thyroid carcinoma
ATC: Anaplastic thyroid carcinoma
MTC: Medullary thyroid carcinoma
WDTC: Well-differentiated thyroid carcinoma
FNAC: Fine needle aspiration cytology
SE (SMD): Standard error of standardized mean difference
VEGF: Vascular endothelial growth factor
131-I: Radioactive iodine
